# Prior math achievement and inventive production predict learning from productive failure

**DOI:** 10.1038/s41539-023-00165-y

**Published:** 2023-05-15

**Authors:** Manu Kapur, Janan Saba, Ido Roll

**Affiliations:** 1 Department of Humanities, Social and Political Sciences, ETH Zürich, Switzerland; 2 Faculty of Education in Science and Technology, Technion, Israel

**Keywords:** Education, Social sciences

## Abstract

A frequent concern about constructivist instruction is that it works well, mainly for students with higher domain knowledge. We present findings from a set of two quasi-experimental pretest-intervention-posttest studies investigating the relationship between prior math achievement and learning in the context of a specific type of constructivist instruction, Productive Failure. Students from two Singapore public schools with significantly different prior math achievement profiles were asked to design solutions to complex problems *prior* to receiving instruction on the targeted concepts. Process results revealed that students who were significantly dissimilar in prior math achievement seemed to be strikingly similar in terms of their inventive production, that is, the variety of solutions they were able to design. Interestingly, it was inventive production that had a stronger association with learning from PF than pre-existing differences in math achievement. These findings, consistent across both topics, demonstrate the value of engaging students in opportunities for inventive production while learning math, regardless of prior math achievement.

## Introduction

Who benefits from constructivist instruction? This concern is at the heart of much debate^1–3^. It is widely agreed that learners give meaning to their experiences. Constructivist theories of learning design further advocate for instructional opportunities that provide learners with explicit opportunities to do so^4,5^. It suggests that students learn by solving authentic, challenging problems^6,7^. Support is provided, for example, from peers or through design elements of the environment and the activities^8^. A constructivist view of learning design supports instruction that challenges learners to engage in sense-making without providing them with ready-made solutions that may short-circuit this process^9–11^. One criticism of this approach is that although challenging problems may work well for students who succeed in solving them (or discover the underlying model), other students may fail to benefit from them^12^. According to this view, to benefit from constructivist activities, one needs to have high-domain knowledge that allows one to navigate their own learning. Research on the Expertise Reversal effect offers one example of the need for novices for increased support^13^.

Research on productive failure (PF) challenges this view^14–16^. PF literature shows that giving students challenging activities on which they fail may, in fact, better prepare them to learn from subsequent instruction, compared with students who do not struggle to solve these challenging problems^15–23^. PF engages students in preparatory problem-solving, where learners activate their general prior knowledge and generate multiple suboptimal solutions, namely, the *inventive production* process, before engaging in subsequent direct instruction^16^. It is argued that the process of struggle offers students valuable experiences with which they can construct meaning of the subsequent instruction^4^.

It is important to mention that different points of view are seen among studies on PF: several studies, such as theses mentioned above, support the PF approach. Other studies support engaging in direct instruction^24,25^ prior to problem-solving. Several studies have compared direct instruction to PF and found no clear benefits to either approach when learning about general inquiry skills^26,27^ and in non-STEM domains^28^. In our study, we are not discussing the benefits of PF over direct instruction or vice versa. Instead, we look into the PF context to explore factors that may contribute to promoting math learning.

This study focuses on inventive production in mathematics education. It explores the relationship between inventive production, prior math achievement (as measured by performance on a standardized problem-solving test), topic-specific prerequisite knowledge (as measured on a pretest), and learning from PF (as measured on a posttest).

To our knowledge, only two studies^29,30^ have illustrated inventive production and its relationship to prior math achievement. They focused on describing the span of strategies that students invented. However, these studies do not highlight which measure better predicts student learning—students’ prior math achievement or their inventive production during the learning process? That is, are students who generate more solutions likely to learn more overall from the subsequent instruction? Although one could conjecture from these studies that prior math achievement may not be strongly associated with inventive production, a quantitative demonstration of the association between prior math achievement and inventive production was lacking.

While most PF studies have been comparative in nature (PF versus Direct Instruction), this paper focuses on student factors *within* the PF design. We evaluate the relationship between inventive production, prior math achievement, and learning from PF. Specifically, we seek to better understand who can benefit from PF instruction, which is constructivist in nature.

### Productive failure

Several studies support the relative effectiveness of engaging in direct instruction1^12,24,25,27^. They argue for providing students with high levels of support (in the form of instruction) prior to problem-solving (referred to as I-PS^15^). For example, the Instruction phase may include a formal introduction to the target domain concepts and work examples. Following, students move to a Problem-Solving phase where they engage in problem solving^31^.

An alternative approach applies the learning sequence of problem-solving followed by Instruction (PS-I^15^). PS-I sequence of learning enables students to participate in solution attempts for problems related to new target concepts prior to the instruction phase that involves lectures and/or practice. The goal of the Problem-Solving phase is to prepare students for future learning from the Instruction phase (PFL^21,22^). When the Problem-Solving and Instruction phases of PS-I are designed in accordance with the principles of PF, the design becomes a PF design^32^. In PF, the design of the problem-solving phase incorporates confronting students with challenging experiences of problem-solving, promoting their agency, and facilitating learning with appropriate cognitive load^32^. Thus conceived, in this study, we focus on the PF design, which is a subset of the PS-I design.

PF^14,32^ is an instructional sequence in which students generate representations and solutions to a novel problem that targets a concept they have not learned yet prior to receiving instruction on the same topic. PF begins with a *generation and exploration phase* in which students are asked to generate and explore the affordances and constraints of multiple Representations and Solution Methods (RSMs). Students are not expected to apply a specific procedure, rather, they are encouraged to develop their own solution approaches. Typically, as demonstrated later in this paper, students apply a wide variety of mathematical tools to design a variety of RSMs. Most of these solutions are not “complete” in terms of their mathematical validity, efficiency, or generalizability. Therefore, the generation and exploration phase is followed by *consolidation and knowledge assembly*, where students learn the targeted concept by organizing students’ representations and ideas based on canonical solutions^16,32^. PF is intentionally designed to result in failure in problem-solving. So, in the following instruction, this process of failure can be productive in preparing students to better learn the target concepts^19^.

Studies have shown that instruction that is based on student-generated RSMs facilitates students’ awareness of specific gaps in their reasoning^33^ and prepares them to learn from subsequent instruction, as suggested by the studies on impasse-driven learning^34^ and test-enhanced learning^35^. In addition, the followed-up instruction, which explicitly emphasizes these gaps and errors, may promote students’ conceptual understanding and learning transfer^14,15^.

### The benefits of inventive production as part of PF

Understanding how students engage with the problem at hand during the generation and exploration phase is important to understand the overall benefit of PF instruction. Schwartz and Martin^22^ use the term *inventive production* to describe the process of generating original solutions to novel problems. In PF instruction, inventive production is based on students’ attempts to generate multiple RSMs during the generation and exploration phases before the instruction phase.

When students attempt to solve a mathematical problem related to the concept they are yet to learn, their attempts to generate multiple RSMs in the Problem-Solving phase potentially have multiple benefits. These include the activation of their general prior knowledge to generate the RSMs, awareness of knowledge gaps when these RSMs are evaluated, and identifying key requirements from the target solution when these failures are then analyzed^15,36^.

Studies^14^ by Watson & Mason (2002) have shown that students have the ability to generate solutions to problems that require concepts they have not been formally taught, albeit these are often partial solutions. In this case, merely engaging in inventive production may be sufficient to prepare students for subsequent instruction (“time for telling”^21^). For example, diSessa and colleagues^37^ found that when sixth graders were asked to invent static representations of motion, students were able to generate and critique a large collection of representations. Likewise, Carpenter and Moser^38^ showed that first-graders, who had not been taught number operations, were able to design different types of strategies for addition and subtraction problems, ranging from rudimentary modeling to more sophisticated strategies. Granberg’s study^39^ explores secondary students’ problem-solving process to solve a linear function problem using a dynamic software program GeoGebra. The findings show that although students constructed incomplete and, in some cases, erroneous new knowledge, most of them have engaged in productive struggle and succeeded in reconstructing useful general prior knowledge and constructing correct new knowledge to solve the problems. In their study on modeling activities, Doerr and English^40^ presented findings that both American and Australian students could devise a number of ranking systems despite not having been formally instructed on the concept.

Similar findings were found in research on model eliciting activities^41^, fractions^42,43^, combinatorial problems^44,45^, number operations^46^, ratios and proportion^47^, and percentages^29^.

These studies make a significant contribution by demonstrating and describing students’ constructive resources^48^ and documenting the possibility space of representations, solutions, and strategies students can generate when given an opportunity to do so. However, these studies do not test the association between inventive production and learning outcomes. For that, we need to examine other studies that have associated inventive production and learning outcomes. For example, Kamii and colleagues^49^ showed that students who designed their own procedures for addition and subtraction demonstrated a better understanding of place value than those who relied on taught algorithms. In a longitudinal study over the course of three years (from grades 1 to 3), Carpenter and colleagues^50^ found that students who invented strategies to solve addition and subtraction problems *prior* to learning the teacher-taught algorithm not only showed better knowledge of the base-ten number concept but were also more successful in solving extension problems than students who relied only on the teacher-taught algorithm. These findings have been extended to mental computation tasks^30,51^ and fractions^52^.

Extending these findings to older children, Schwartz and Martin^22^ showed that ninth-grade students who invented an index of variance before a lecture on the topic outperformed the comparison group on transfer measures. Similarly, Levav-Waynberg and Leikin^53^ reported that tenth-grade students who attempted to prove new geometrical theorems over the course of a year developed expertise and enhanced the connectedness of their geometrical knowledge, compared to a comparison group of students who had not any special intervention. Terwel and colleagues^54^ showed that students who learned to use representation in the process of collaborative design outperformed their peers who were taught the target representation in a more traditional way. Kapur^32,55^ reported a positive correlation between the number of RSMs generated during PF and conceptual knowledge acquisition.

However, results on the relationship between inventive production and learning are mixed. Two studies, that used the same learning materials, found that in the PF condition, students’ general prior knowledge activation (measured by the number and quality of RSMs) did not correlate significantly with their performance on the conceptual knowledge post-test^33,56^.

Taken together, the abovementioned studies suggest that: (a) students have the constructive resources to invent solutions to the novel, challenging problems that target concepts they have not learned yet, and (b) mixed results were found related to the benefit of instruction that engages students in inventive production for math learning. What is missing from the above review is evidence that associates students’ prior math achievement, inventive production, and learning outcomes.

As the PF process depends on students’ engagement in inventive production, we are interested in examining the key factor that influences inventive production, which is prior math achievement. Lembke and Rey’s study^29^ took into account students’ prior math achievement as measured by a standardized problem-solving test and showed that average-ability students could invent almost the same number of strategies as high-ability students. Heirdsfield^30^ also reported on a group of low-ability students who were able to invent strategies as a way to compensate for less knowledge. Although one could conjecture from these two studies that prior math achievement may not be strongly associated with inventive production, a quantitative demonstration of the association between prior math achievement and inventive production was lacking.

In this paper, we examine the associations between prior math achievement, inventive production, and learning outcomes from PF. We operationalize this using two research questions:

i. What is the relationship between students’ prior math achievement and their ability to engage in inventive production, that is, generate novel solutions during problem-solving?
ii. Which measure better predicts student learning from PF—students’ prior math achievement, students’ topic-specific prerequisite knowledge, or their inventive production during the learning process?

Our first conjecture is that inventive production may not depend as strongly on prior math achievement as one would expect. Our second conjecture is that learning from PF does not depend as much on prior math achievement or topic-specific prerequisite knowledge as it does on students’ inventive production

To identify the relationship between prior math achievement, inventive production, and learning from PF outcomes, we investigated empirical evidence from a set of studies in Singapore math classrooms. The studies focused on two “big ideas” in math education that are often conceptually challenging: (a) ratios (specifically, average speed) for seventh-grade students and (b) variance (specifically, standard deviation) for eighth-grade students. To strengthen the external validity of the study, we chose two topics that are sufficiently unrelated to each other.

Students from two schools, hereinafter referred to as Schools A and B, were selected based on the academic ability profile of their student intake as evidenced by the primary school leaving examination (PSLE). The PSLE is a sixth-grade national standardized examination based on Singapore’s curricular and content standards for Mathematics, English, Science, and Mother Tongue. The aggregate score on the PSLE forms the major criteria used to enter secondary schools (i.e., grades 7–10) in Singapore.

Table 1 presents the descriptive statistics for the PSLE math grade and PSLE total score for the two schools. MANCOVA was conducted with the PSLE math grade and PSLE total score as the two dependent variables. Results of analyzing seventh-grade students’ scores revealed a significant multivariate effect between the two schools, *F*(2, 109) = 282.97, *p* < 0.001. Compared to students from School B, students from School A achieved significantly higher PSLE scores, *F*(1, 110) = 464.42, *p* < 0.001, *d* = 5.34, and PSLE math grade, *F*(1, 110) = 110.44, *p* < 0.001, *d* = 2.12. Similarly, results of analyzing eighth-grade students’ scores revealed a significant multivariate effect among the two schools, *F*(2, 102) = 76.26, *p* < 0.001. Compared to students from School B, students from School A achieved significantly higher PSLE scores, *F*(1, 103) = 154.02, *p* < 0.001, *d* = 2.49, and PSLE math grade, *F*(1, 103) = 35.47, *p* < 0.001, *d* = 1.23.

**Table 1:** Descriptive statistics for PSLE performance between Schools A and B.

			PSLE math grade (lower is better)		PSLE total score (higher is better)	
Ratios study (seventh grade)	School	N	M	SD	M	SD
	School A (higher-performing students)	36	1.11	0.32	235.4	3.01
	School B (lower-performing students)	76	2.26	0.62	211.2	6.40
Variance study (eighth grade)	School A (higher-performing students)	69	1.74	0.63	233.4	9.34
	School B (lower-performing students)	36	2.64	0.89	211.5	6.64

The studies on both topics were quasi-experimental with a pretest-intervention-posttest design. Each study was carried out as part of regular curriculum time. One week before the start of each study, all students took a 30-min pretest as a measure of topic-specific prerequisite knowledge of the targeted concept. Seventh-grade students completed an 8-item pretest (*α* = 0.72) on the prerequisite concepts of speed, average speed, and rate of change. Eighth-grade students took a five-item pretest (*α* = 0.75) on the prerequisite concepts of central tendencies and distributions, and variance.

While prior math achievement, as evaluated by the PSLE, evaluates overall knowledge of math and its application, the pretests (which assess students’ topic-specific prerequisite knowledge) measure relevant prerequisite knowledge rather than specific knowledge of the targeted topics (see “Data Collection” section).

In both studies, the PF instruction was delivered in two phases—the generation and exploration phase and the consolidation phase^16,32^. In the generation and exploration phase, which lasted two periods, Students were assigned into groups (triads) by the teacher based on teachers’ knowledge about their students. The choice of working within groups (a key PF fidelity criterion^16^) is based on vicarious failure (VF), which addresses that observing other students’ failure to solve a problem would also be productive for her\his own learning from subsequent instruction^56,57^. Studies illustrate the benefit of exposing students to other’s general prior knowledge and expertise in developing, detecting, and correcting multiple RSMs^58^. Findings show that not all students in the generation and exploration phase have to generate solutions themselves; By observing their classmates’ generation solution process, they can obtain equal preparation for learning^56^ and activate their general prior knowledge similar to their partners^59^. In our study, through group discussions, students designed solutions to solve a complex problem involving the targeted concepts (see supplementary materials for the complex problem). During this phase, no extra support or scaffolds were provided, nor was any homework assigned.

During the consolidation phase, the teacher asked the groups to share their RSMs, that is, their invented solutions, with the goal of comparing and contrasting the affordances and constraints of the student-generated RSMs. The teacher then shared the canonical ways of solving the problems with the class. While doing so, the teacher drew comparisons and contrasts between the canonical and student-generated RSMs and, in the process, explicated the targeted concept in the context of the problems. Finally, students practiced solving isomorphic problems, and the teacher discussed the solutions to these problems.

The consolidation phase was given to the entire class as a whole and thus had no between-group variability. Following the instruction, students practiced applying the taught procedure. It is important to emphasize that both groups in the two studies received the same testing procedure and the same instructional manipulation.

One week after the unit, all students took a posttest (which was not equivalent to a pre-test) as a measure of their learning. Seventh-grade students completed a 35-min, 5-item posttest (*α* = 0.78) after the study. Eighth-grade students completed a 45-min, six-item posttest (*α* = 0.78). Both posttests comprised three items on procedural fluency, two items on conceptual understanding, and one item on near transfer.

To evaluate inventive production, the artifacts (the RSMs) that were generated by the groups of students were analyzed. We used Kapur and colleagues’ measure of the total number of different RSMs generated by a group^18,32^. We acknowledge that the number of RSMs may be a simplistic measure of inventive production. However, the number of RSMs is a practical measure that does not introduce bias.

## Results

Tables 2 and 3 present the descriptive statistics for the pretest scores, number of RSMs, and posttests scores for Schools A and B in each of the topics: ratios unit and variance unit, respectively.

**Table 2:** Descriptive statistics for seventh-grade students’ performance in ratios unit in Schools A and B.

		School A (*n* = 36)		School B (*n* = 76)	
Measure	Max	M	SD	M	SD
Ratios-pretest (topic-specific prerequisite knowledge)	10	8.60	0.85	8.89	1.53
No. of RSMs	9	6.83	1.44	6.16	1.38
Ratios-posttest	10	7.82	1.36	6.59	1.75

**Table 3:** Descriptive statistics for eighth-grade students’ performance in variance unit in Schools A and B.

		School A (*n* = 69)		School B (*n* = 36)	
	Max	M	SD	M	SD
Variance-pretest (topic-specific prerequisite knowledge)	10	8.16	1.82	8.35	1.36
No. of RSMs	9	5.23	1.50	5.19	1.49
Variance-posttest	10	6.00	2.02	5.45	2.12

### Pretests

An ANOVA did not reveal any significant difference between the two schools on their topic-specific prerequisite knowledge: For school A: *M* = 8.60, *SD* = 0.85, for school B: *M* = 8.89, *SD* = 1.53, *F*(1, 110) = 1.530, *p* = 0.219 in the pretest on ratios. For school A: *M* = 8.16, *SD* = 1.82, for school B: *M* = 8.35, *SD* = 1.36, *F*(1, 103) = 0.303, *p* = 0.583 in the pretest on the topic of variance. It is important to notice that in both studies, both schools had similar high scores in the topic-specific pre-tests (see Table 3). These results indicate that both schools have similar relevant topic-specific prerequisite knowledge of the target concepts.

### Inventive production (number of RSMs)

As students were not familiar with the target concepts of ratios and variance, they applied a variety of approaches and heuristics, such as qualitative analysis, algebraic approaches, and trial-and-error, to name a few. Notably, no single method was likely to solve the given challenge. In fact, none of the groups successfully solved the given problem during the generation and exploration phase. Instead, students were encouraged to persist in exploring the design space. Overall, for each unit (ratios and variance), we identified nine different RSMs in students’ written work. The full span of RSMs is detailed in the supplementary materials.

To test our first conjecture, we examined the effect of prior math achievement (by sampling students from schools with significantly different PSLE math grades) on inventive production. An ANOVA revealed a significant difference between the two schools on the number of RSMs in ratio unit, for school A: *M* = 6.83, *SD* = 1.44, for school B: *M* = 6.16, *SD* = 1.38, *F*(1, 110) = 5.669, *p* = 0.019, *d* = 0.48. In variance unit. An ANOVA did not reveal any significant difference between the two schools on the number of RSMs, for school A: *M* = 5.23, *SD* = 1.50, for school B: *M* = 5.19, *SD* = 1.49, *F*(1, 103) = 0.015, *p* = 0.903, *d* = 0.03.

### Posttests

To test the second conjecture, we analyzed the effects of prior math achievement (measured by PSLE math grade), topic-specific prerequisite knowledge (measured by pretest scores), and inventive production (measured by the number of RSMs) on posttest performance while accounting for the effects of school. Therefore, we carried out an ANCOVA with posttest score as the dependent variable, school as the between-subjects factor, PSLE math grade, topic-specific prerequisite knowledge, and number of RSMs as the three covariates. The analysis of the ratios-posttest revealed that both the number of RSMs, *F*(1, 107) = 62.589, *p* < 0.001, and PSLE math grade, *F*(1, 107) = 4.436, *p* = 0.032, had significant effects on the posttest performance. Topic-specific prerequisite knowledge, *F*(1, 107) = 2.725, *p* = 0.102, and school, *F*(1, 107) = 0.522, *p* = 0.471, did not. However, The analysis of the variance-posttest revealed a significant effect only on the number of RSMs, *F*(1, 100) = 105.518, *p* < 0.001. PSLE math score, *F*(1, 100) = 0.001, *p* = 0.980, school, *F*(1, 100) = 2.394, *p* = 0.125, or Topic-specific prerequisite knowledge, *F*(1, 100) = 0.493, *p* = 0.484, we not associated with posttest scores.

## Discussion

This study sought to evaluate the relationship between students’ incoming knowledge and their learning from PF instruction. We identified two main findings:

First, a weak-to-no association between prior math achievement and inventive production. results of the variance unit show no significant difference between students from the school with higher prior math achievement and those from the school with lower prior math achievement. Results from the ratios unit show that 7th-grade students from the school with higher prior math achievement demonstrated significantly better inventive production than those from the school with lower prior math achievement. While an effect size of nearly .5 is considered large, it should be examined in relation to the overall difference between schools. To put it into context, the effect size difference between Schools A’s and B’s students on their inventive production (*d* = 0.48) was less than a quarter of their pre-existing difference in their prior math achievement (*d* = 2.12, see Sample section above). These results seem to suggest that inventive production may not depend as strongly on general prior math achievement as one would expect. However, it is important to note that the topic-specific prerequisite knowledge was similar across schools. Thus, while there were broad differences in prior math achievement, these differences were smaller about prerequisite concepts, which may explain the smaller difference in inventive production.

Second, we found that the association between inventive production and learning from PF was much stronger than that of pre-existing differences in prior math achievement in both topics. Prior math achievement was not associated with posttest scores in the variance unit, and was associated with posttest in the ratios unit, though to a much lesser degree. Topic-specific prerequisite knowledge had no association with learning on both posttests results. We explain both findings.

Weak-to-no association between prior math achievement and inventive production. Sinha and Kapur (2021) address that engaging students in preparatory problem-solving allows them to maximally activate their knowledge and generate new suboptimal solutions, which in turn prepare them for the following subsequence of direct instruction. Our findings can take this claim one step further and suggest that students’ prior math achievement does not play a critical role in their execution in inventive production. While the two schools differed significantly in their prior math achievement, results from the study on the two topics revealed that students were able to generate and design a similar number of RSMs for each unit. This supports our conjecture that students who were vastly different in their general prior math achievement were not as different in their inventive production as one would expect, given the prior math achievement differences. While Lembke and Reys^29^ and Heirdsfield^30^ appeared to have similar findings, theirs were anecdotal and descriptive. Our study not only demonstrates but also produces empirical evidence to support this. Thus, the answer to Research Question 1 is that while there is some association between prior math achievement and inventive production, this is not nearly as strong as one may expect. Student groups who are very different in their prior math achievement were much closer in their inventive production.

This result is somewhat surprising, as inventive production depends on general prior knowledge, and the two schools had very different mathematical backgrounds. Why did the superior math knowledge of students in School A not help them to be much more inventive during the generation process? One possible explanation could be that mathematics instruction simply does not require students to be inventive or generative, and therefore, students of different prior math achievements have had similar opportunities to practice (and develop expertise in) inventive production. Alternatively, prior math achievement requires different properties of knowledge compared with inventive production. Students who are excellent problem solvers possess a highly-organized, easily accessible knowledge base that allows them to search the solution space efficiently, automatically triggering possible solution paths^60^. However, when engaging in inventive production, students are unable to apply the same strategy, as these require engagement in divergent search and generating solutions outside students’ scope of expertise. Another explanation could be that it is hard for students to use their formal math knowledge to generate solutions to novel challenges. The transfer is often rare, and without appropriate prompting, students may have failed to transfer their knowledge. However, the PF activity was designed to activate general prior knowledge^16^.

Finally, an important feature of PF is that progress can be made using intuitive ideas and can be evaluated using the given data^15^. The more formal knowledge of students in School A may have been less relevant to this kind of task. However, as students in both schools covered similar curricula (albeit at different levels), also students in School B had access to the same knowledge resources that fed into their invented methods.

Inventive production was more strongly associated with learning from PF than pre-existing differences in math achievement. Mixed results were addressed in the literature review related to the association between inventive production and learning from PF. The results of this study are not in alignment with Loibl and Rummel^33^ and Hartmann et al^56^. works, who found no association between inventive production (tested by the number and quality of RSMs) and learning from PF. However, our study is similar to other studies that have associated inventive production and learning from PF outcomes^22,52,54^. Our results revealed that invention production had a very strong relationship with learning from PF; that is, the greater the number of RSMs generated, the better the learning from PF outcomes. Furthermore, the number of RSMs was by far the main factor influencing learning from PF outcomes of the factors that were measured in the current study; prior math achievement had only a small effect (in the topic of ratios) or no effect (in the topic of variance) on learning outcomes.

Topic-specific prerequisite knowledge, too, did not have any significant effect on learning from PF. This result is in alignment with Hartmann’s et al. study^56^. Results of their study show that there was only a significant difference between VF (VF: observing other students’ failing to solve a problem) and PF conditions for students who had a certain amount of topic-specific prerequisite knowledge. While topic-specific prerequisite knowledge did not affect the post-test performance in the PF condition.

These results validate an important characteristic of PF that has not until now been examined: the degree to which students benefit from these learning activities does not depend as much on their prior math achievement as it does on what they generate during the initial problem-solving. Put differently, the criticism that suggests that only students who succeed in the inventive production activity, namely inventing correct RSMs, learn is inaccurate—not only that all students fail to generate the correct solution, but also learning from PF does not depend as much on topic-specific prerequisite knowledge or prior math achievement.

The question which could be raised is why inventive production is strongly associated with math learning in PF instruction? we propose several interdependent mechanisms. First, as mentioned earlier, engaging in inventive production may be better at activating and differentiating relevant general prior knowledge, provided students are able to use their priors to generate sub-optimal or even incorrect solutions to the problem^61–64^. Thus, knowledge activation prepares learners to learn from subsequent instruction^34,35^. Second, general prior knowledge activation may, in turn, afford more opportunities for students to: (a) notice the inconsistencies in and realize the limits of their general prior knowledge^61,65,66^, and (b) compare and contrast student-generated solutions and correct solutions during subsequent instruction, thereby helping students to attend to and better encode critical features of the new concept^19,63^. Finally, besides the cognitive benefits, problems such as the ones given during the generation and exploration phase may also have affective benefits of greater learner agency, as well as engagement and motivation to learn the targeted concept^67,68^.

### Limitations

One limitation of our study has to do with the population, the topics studied, and the teachers. The study contrasted high- and medium-level schools related to students’ prior math achievement and thus may not extend to the lower end of the spectrum or to other topics. Different teachers taught in the two schools, this also could be one limitation of our study. However, the instruction phase was similar in both groups. The fact that the effects are very consistent across schools suggests that it is not teachers (random effect) but rather instruction.

Another limitation stems from within-school variability. While the generation phase took place in groups, students’ prior math achievement was measured individually using the PSLE. It is possible that the lack of correlation between PSLE scores and the number of RSMs is due to the fact that weak group members were credited with RSMs that were developed by high-ability group members.

However, it is important to emphasize that in relation to prior math achievement, the within-school variability (and hence, within-group variability) was much lower than between-school variability. Future work should further investigate the effect of group composition on the number of RSMs. In addition, the PSLE results indeed reflect different aspects of knowledge (e.g., familiarity with math concepts, problem-solving, attitudes towards math, etc.). It is thus somewhat unclear what explains the association between high PSLE scores and a high number of RSM. Yet, the study found only weak associations and only one topic. Thus, PSLE scores, while a composite of different math-ability aspects, does not offer a strong explanation for the variability of RSMs.

Finally, because students worked in groups during the initial problem-solving phase, there is a clear nesting of the data. Ideally, we would have liked to have used multi-level modeling. However, we did not have a large enough number of groups to reliably estimate the parameters. Therefore, in such instances, to test for the independency of data obtained in group settings, Kenny, Kashy, and Bolger^69^ suggest the calculation of intra-class correlations (ICC) to test for consequential non-independence. Because the ICC for group members’ individual posttest scores was not significant in both topics of the study, it was acceptable to analyze learning from PF outcomes on an individual level.

## Conclusion

Constructivist instruction offers many intriguing benefits in the form of deep conceptual understanding through authentic problem-solving. However, a common concern is that only better students benefit from such instruction. Here we studied in-depth one type of constructivist instruction, Productive Failure. Our findings suggest that there is potential for activities that require inventive production to narrow the achievement gap one would expect due to initial differences in prior math achievement. We do not claim that our findings will hold true more generally, much less speak to the problem of the achievement gap in other countries. What we do have evidence for is that starting with students with significantly different prior math achievements, we were able to demonstrate how engaging them in inventive production was able to reduce the gap between them in the learning of mathematical content. Our findings offer exciting opportunities in that student from different backgrounds can achieve similarly high learning from PF gains. They show that built correctly, instruction can help narrow the social gap and give opportunities to all learners to develop math expertise. As educators and researchers, it is our obligation to further explore this promise.

Overall this study makes several contributions. Theoretically, it contributes to the literature on PF as it emphasizes the critical role which inventive production can play in narrowing the gap between students with diverse math backgrounds. Findings showed that productive invention (creating more RSMs to a given problem) promotes learning regardless of prior math achievement. Prion math achievement is not associated with inventive production. Furthermore, this study contributes to the vicarious learning literature by showing the association between exposure to RSMs at the group level with learning at the individual level. Pedagogically, this study suggests facilitating PF learning environments that emphasize and give more space to inventive production to encourage students to activate their prior knowledge and create more RSMs for the problems. This kind of emphasis may significantly contribute to promoting math learning.

## Methods

### Participants

One hundred and twelve seventh-grade students and 105 eighth-grade students from two mainstream, coeducational public schools in Singapore participated in this set of studies. The medium of instruction throughout the Singapore school system is English. Students at these schools typically come from middle-class socioeconomic backgrounds. The two schools, hereinafter referred to as Schools A and B. As mentioned earlier in this paper, students from School A achieved significantly higher PSLE scores and PSLE math grades than students from School B. Thus, students from School A have better prior math achievement compared to students from School B. The methods were performed in accordance with relevant guidelines and regulations. IRB approval of the National Institute of Education, Singapore, for this research was obtained; and the procedures duly followed. Written informed consent to take part in the study was obtained from parents and oral consent from children, who acknowledged that they were free to withdraw at any time without penalty.

### Data collection

#### Pretest

(1) Ratios-pretest consisted of eight items: three items on speed, three items on the rate of change, and two items on average speed (see supplementary materials); (2) variance-pretest consisted of four items: two items test prerequisite concepts of central tendencies and 2 items related to distributions (see Supplementary materials).

#### Group work artifacts and discussions

The artifacts that were generated by the groups were used to evaluate their inventive production, as detailed below. Each group of students was given blank sheets of A4 paper for their group work. All group discussions were captured in audio and transcribed by a research assistant

#### Posttest

(1) Ratios-posttest included five items that targeted students’ ability to identify and use relevant critical features and information to solve problems at average speed (see Supplementary materials). (2) Variance-posttest included six items that target students’ ability to identify and use relevant critical features and information to solve problems on variance (see Supplementary materials).

### Data analysis

#### Pretests

Solutions of the pretests were scored as incorrect (0 points), incomplete solutions with correct representational and strategy deployment (1 point), partially correct solutions that demonstrated correct representational and strategy deployment but computational errors (2 points), or fully correct (3 points). Although several students in the ratios pretest was able to solve speed and rate of change items, none of them were able to solve the two average speed items, which evidenced the fact that the concept was indeed novel to them. Hence, the two items on average speed were not included in the pretest composite score. To allow for ease of comparison, the composite pretest score (maximum of 18 points in ratios pretest and maximum of 12 points in variance pretest) was scaled (linearly) to have a maximum of 10 points.

#### Inventive productive

To determine the total number of RSMs generated by each group, we analyzed the group work artifacts and the discussion transcripts using the analytical scheme that Kapur and colleagues have developed and reported on^18,32^. Briefly, the RSMs identified in the group work artifacts were used to segment the group discussion into smaller episodes. For example, if the group work artifacts revealed that the group used ratios to solve the problem, then the relevant episode from the discussion in which the group discussed the ratios method was identified. An episode started with the first proposal of a new RSM and ended when the group either abandoned it or moved on to another RSM. Segmenting of a discussion into episodes was simplified by the fact that there were generally clear transitions in the discussions when a group moved from one RSM (e.g., ratios) to another (e.g., algebra). Analysis was focused solely on RSMs, and episodes of non-task behavior and social talk were not included in the analysis. This process was repeated for all PF groups.

#### Posttests

Similar to the pretest data analysis, posttests solutions were scored in the same manner as incorrect (0 points), partially correct (1 or 2 points), or fully correct (3 points). For ease of comparison, the composite score on the posttest (maximum of 15 in ratios-posttest, and maximum of 18in variance-posttest) was scaled (linearly) to have a maximum of 10 and formed the dependent variable in our analyses.

### Validity and reliability

The pretests and the posttests were designed according to Singapore’s national curricular and mathematical content standards for both units. The pretest and posttest were reliable measures of students’ knowledge, with Cronbach (ratios: pretest, *α* = 0.72; posttest, *α* = 0.77; variance: pretest, *α* = 0.78; posttest, *α* = 0.78). Two experienced raters independently scored students’ solutions with inter-rater reliability Krippendorff’s alpha (ratios: 0.95 in the pretest and 0.87 in the posttest; variance: 0.98 in the pretest and 0.96 in the posttest). All disagreements were resolved via discussion with the first author. For inventive production, two raters independently segmented the group transcripts into episodes and coded the episodes into RSM type. The inter-rater reliabilities (Krippendorff’s alphas) for segmenting transcripts into episodes and coding of the episodes were 0.94 and 0.97 (ratios unit) and 0.94 and 0.95 (variance unit), respectively for this study.

The pre-and post-tests provide scores at the individual level. However, the inventive production measure provides input at the group level. We chose to keep this measure for two reasons. First, from a theoretical perspective, we sought to quantify the number and diversity of solutions with which students engaged. As shown before, students may learn from VF as much as they learn from failing on their own^56^. Moreover, these solution approaches emerged from the group discussion and cannot be attributed to any individual member. Thus, the group-level variable is a good approximation of the solutions with which each group member engaged. Second, from an applied perspective, given the size of the groups, we did not find a relevant statistic (such as HLM) that could model the nesting of individual learners within groups. That being said, analysis at this level may create a dependency between data points within groups. To test for the independency of data obtained in group settings, Kenny, Kashy, and Bolger^69^ suggest the calculation of ICC to test for consequential non-independence. The ICC of posttests scores was not significant, allowing us to analyze learning from PF outcomes on an individual level.

### Reporting summary

Further information on research design is available in the Nature Research Reporting Summary linked to this article.

## Supplementary information

Supplementary Material

Reporting Summary

## Data Availability

The datasets used and/or analyzed during the current study are available from the first author upon reasonable request. The materials are available in the supplementary materials.
