# Supplementary material for: Prior math achievement and inventive production predict learning from productive failure
Source: NPJ Sci Learn. 2023 May 15;8:15. doi: 10.1038/s41539-023-00165-y (PMC10185511; doi:10.1038/s41539-023-00165-y)
Supplement: Supplementary file 1 — Supplementary Material [file 41539_2023_165_MOESM1_ESM.docx]

# Supplementary Materials

1. Complex Problem for Average Speed Unit 2
2. Example of items on the Average Speed Pretest 4
3. Example of items on the Average Speed Posttest 5
4. Complex Problem for Variance Unit 7
5. Example of items on the Variance Pretest 8
6. Examples of items on the Variance Posttest 10
7. Student-generated RSMs to the Average Speed Problem 11
8. Student-generated RSMs to the Variance Problem 15

1. Complex Problem for Average Speed Unit

It was a bright, sunny morning and the day of the Singapore Idol auditions. Hady and Jasmine were going to audition as a team. They were practicing at their friend Ken’s house and were planning to bike to the auditions at Singapore Expo. The auditions were supposed to start at 2 pm and Hady and Jasmine wanted to make sure that they could make it in time.

Hady: “Ken, how do we get to the Singapore Expo from here?”

Ken: “Well, follow this road (pointing to a map) until you reach the expressway. I usually drive at a uniform speed of 90 km/h on the expressway for about 3 minutes. After that there is a sign telling you how to get to Singapore Expo.”

Jasmine: “How long does it take you to reach Singapore Expo?”

Ken: “It normally takes me 7 minutes to drive from my house when I am

traveling at an average speed of 75 km/h.”

After getting the directions, Hady and Jasmine left Ken’s house and biked together at Jasmine’s average speed of 0.15 km/min. After biking for 25 minutes, Jasmine biked over a piece of glass and her tire went flat.

Jasmine: “Oops! My tire is flat! What shall we do now? Can I just ride with you on your bike or shall we take a bus the rest of the way?”

Hady: “I don’t think that is a good idea. My bike is old and rusty and it cannot hold both of us. Taking the bus is not a very good idea either. There is no direct bus from here to Singapore Expo, so we would have to take one bus and then transfer to another one. All the waiting for buses would definitely make us late. Do you have any money on you?”

Jasmine: “Let me check….. I forgot to withdraw money today. I only have $2.”

Hady: “I did not bring my wallet. I only have $1 for a drink.”

Jasmine: “Since we do not have enough money to take a taxi, shall we just leave our bikes here and walk?”

Hady: “It takes me approximately 5 minutes to walk to school which is about 250 meters from my home. How long does it take you to walk to school?”

Jasmine: “It takes me approximately 14-16 minutes to walk to school which is about 450 meters from my home.”

Hady: “No, no, no! Walking would take too much time. We will end up late. Why don’t you lock up your bike and take my bike and bike ahead. Leave my bike somewhere along the route and begin walking to the audition. I will walk from here until I get to my bike and ride it the rest of the way since I can bike at a faster speed. My average biking speed is 0.2 km/min.”

Jasmine: “That sounds like a good idea! But how far should I ride your bike before leaving it for you and walking the rest of the way. Since we are auditioning together as a team, we have to reach there at the same time!?”

How far should Jasmine ride Hady’s bicycle so they both arrive at the audition at the same time? Please explain and show all your work.

2. Example of items on the Average Speed Pretest

**Example Item 1**

Mr Tan took 40 minutes to drive from his home in Clementi to the airport at an average speed of 60 km/h. How far is the airport from his home?

**Example Item 2**

To travel the first 50km, you take 30 min. To travel the next 50 km, you take 40 min. What is your average speed for the entire 100km journey?

3. Example of items on the Average Speed Posttest

**Example Item 1**

David travels at an average speed of 4 km*/*hr for 1 hour. He then cycles 6 km at an average speed of 12 km*/*hr. Calculate his average speed for the entire journey.

**Example Item 2**

Hummingbirds are small birds that are known for their ability to hover in mid-air by rapidly flapping their wings. Each year they migrate approximately 9,000 km from Canada to Chile and then back again. The Giant Hummingbird is the largest member of the hummingbird family, weighing 18– 20 gm. It measures 23 cm long and it flaps its wings between 70–80 times per minute. For every 18 hours of flying it requires 6 hours of rest. The Broad Tailed Hummingbird flaps its wings 100–125 times per minute. It is approximately 10–11 cm long and weighs approximately 3–4 gm. For every 12 hours of flying it requires 12 hours of rest. If both birds can travel 1 km for every 550 wing flaps and they leave Canada at approximately the same time, which hummingbird will get to Chile first?

**Example Item 3**

Bob drove 140 miles in 2 hours and then drove 150 miles in the next 3 hours. Study the two speed–time graphs A and B carefully. Which graph—A, B, or both—can represent Bob’s journey?

| **Graph A**    **Speed** | **Graph B**    **Speed** |
| --- | --- |

This item was adapted from Stanford Research International’s research on SimCalc and the Math of Change

4. Complex Problem for Variance Unit

Mr. Fergusson, Mr. Merino, and Mr. Eriksson are the mangers of the Supreme Football Club. They are on the lookout for a new striker, and after a long search, they short-listed three potential players: Mike Arwen, Dave Backhand, and Ivan Right. All strikers asked for the same salary, so the managers agreed that they should base their decisions on the players’ performance in the Premier League for the last 20 years. Supplementary table 1 below shows the number of goals that each striker had scored between 1988 and 2007.

*Supplementary table 1*. Number of goals scored by three potential strikers in the Premier League

| **Premier League Year** | **Mike Arwen** | **Dave Backhand** | **Ivan Right** |
| --- | --- | --- | --- |
| 1988 | 14 | 13 | 13 |
| 1989 | 9 | 9 | 18 |
| 1992 | 14 | 16 | 15 |
| 1993 | 10 | 14 | 10 |
| 1994 | 15 | 10 | 16 |
| 1995 | 11 | 11 | 10 |
| 1996 | 15 | 13 | 17 |
| 1997 | 11 | 14 | 10 |
| 1998 | 16 | 15 | 12 |
| 1999 | 12 | 19 | 14 |
| 2000 | 16 | 14 | 19 |
| 2001 | 12 | 12 | 14 |
| 2002 | 17 | 15 | 18 |
| 2003 | 13 | 14 | 9 |
| 2004 | 17 | 17 | 10 |
| 2005 | 13 | 13 | 18 |
| 2006 | 18 | 14 | 11 |
| 2007 | 14 | 18 | 10 |
| 2008 | 19 | 14 | 18 |
| 2009 | 14 | 15 | 18 |

The managers agreed that the player they hire should be a consistent performer. They decided that they should approach this decision mathematically, and would want a formula for calculating the consistency of performance for each player. This formula should apply to all players and help provide a fair comparison. The managers decided to get your help.

Please come up with a formula for consistency and show which player is the most consistent striker. Show all working and calculations on the paper provided.

5. Example of items on the Variance Pretest

**Example Item 1**

The timings (in minutes) for a 2.4 km run for 40 students in Class 2E1 are shown below. Calculate the mean, median and mode of the timing of Class 2E1.

11; 11; 12; 12; 12; 12; 13; 13; 13; 13; 13; 14; 14; 14; 14; 14; 14; 15; 15; 15; 15; 15; 15; 15; 15; 15; 15; 15; 16; 16; 16; 16; 16; 16; 16; 16; 17; 17; 17; 17

**Example Item 2**

The heart rate per minute of a group of 20 adults is displayed in the dot diagram below. For example, 3 adults have a rate of approximately 50 beats per minute. Based on this data set, how many individuals from a similar group of 40 adults would be expected to have a heart rate of at least 80 beats per minute?

Dot diagram for the heart rate per minute for a group of 20 adults

|  | |  | |  | | ⚫ | |  | |  | |  | |
| --- | --- | --- | --- | --- | --- | --- | --- | --- | --- | --- | --- | --- | --- |
|  | |  | |  | | ⚫ | |  | |  | |  | |
|  | |  | | ⚫ | | ⚫ | |  | |  | |  | |
|  | | ⚫ | | ⚫ | | ⚫ | | ⚫ | |  | |  | |
|  | | ⚫ | | ⚫ | | ⚫ | | ⚫ | | ⚫ | |  | |
| ⚫ | | ⚫ | | ⚫ | | ⚫ | | ⚫ | | ⚫ | | ⚫ | |
|  |  |  |  |  |  |  |  |  |  |  |  |  |  |
|  |  |  |  |  |  |  |  |  |  |  |  |  |  |
| 40 | | 50 | | 60 | | 70 | | 80 | | 90 | | 100 | |
|  | |  | |  | |  | |  | |  | |  | |

6. Examples of items on the Variance Posttest

**Example Item 1**

Marks scored by 10 students on a test on statistics are shown below. As a measure of the variance, calculate the *standard deviation* of the test scores above.

30, 60, 50, 60, 55, 50, 90, 80, 70, 60

**Example Item 2**

For the previous question, one student came up with another measure of variance by taking the *average of the sum of the difference between adjacent scores* as shown below:

= 3.33

How does the student’s measure of variance compare with the standard deviation as a measure of variance? Which one is better? Please explain your answer.

**Example Item 3**

A set of data consists of the following five numbers:

0, 3, 4, 6, 12

I want to add two numbers to this data to create a set of seven numbers without changing the mean and standard deviation. Which two numbers can I add?

## 7. Student-generated RSMs to the Average Speed Problem

A total of nine different RSMs emerged from this analysis (see Supplementary figures 1 and 2):

**1. Hady should walk more*.*** All groups were able to develop the idea that because Hady’s biking speed was greater than Jasmine’s, he should do more of the walking. This was a qualitative concept that emerged in the group discussions.

**2. Jasmine’s walking distance must equal Hady’s biking distance and vice versa.** All groups went further to generate the insight that the partitioning of the total distance into walking

| **Lowest Common Multiple (LCM) RSM** | **Highest Common Factor (HCF) RSM** |
| --- | --- |
| 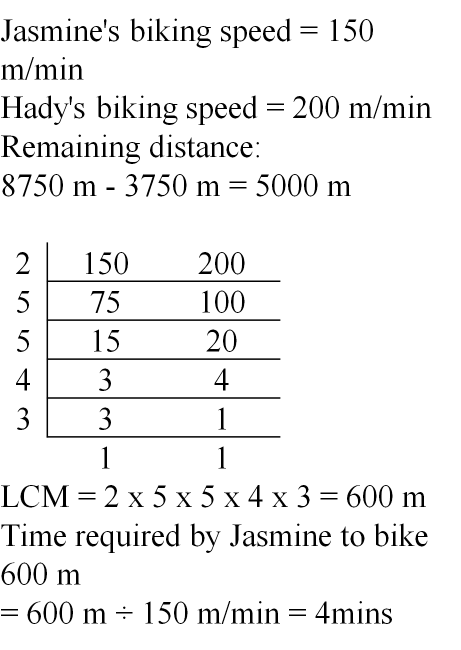 | 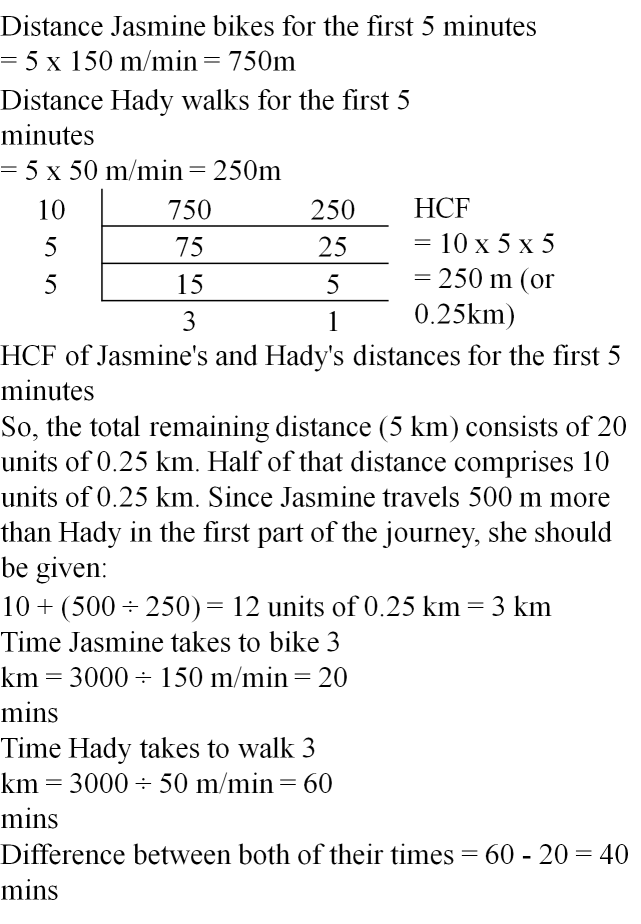 |

*Supplementary figure 1*. Examples of the LCM and HCF RSMs. RSM = representation and solution method. Actual RSMs produced by students were converted into stylized examples for easier reading.

and biking components should be such that Jasmine’s walking distance must equal Hady’s biking distance and that Jasmine’s biking distance must equal Hady’s walking distance.

**3. Diagrams.** All groups were able to draw an accurate diagram to represent the journeys of Jasmine and Hady. These diagrams contained information about distances, speeds, and partition point. For all groups, the diagrams seemed to anchor their problem-solving efforts. An example of such diagrammatic representations can be found in Supplementary figure 2.

**4. Least Common Multiplier (LCM) / Highest Common Factor (HCF).** Some groups used their prior knowledge of LCM and HCF to represent and solve the problem. Referring to Supplementary figure 1, one group took the LCM of the biking speeds to determine the shortest distance into which the biking speeds would factor, including the time it would take to do so. However, the group did not pursue this method any further, in part because the LCM did not form a proper factor of the distance to be traveled (i.e., 600 does not divide 5,000 completely). The same group then tried to find the HCF of Jasmine’s biking speed and Hady’s walking speed. Again, the strategy was to find the number of times the HCF divided the remaining distance and then apportion the parts accordingly. This method too did not lead to a successful solution.

**5. Ratios.** The use of ratios or proportions was fairly common (see Supplementary figure 2). The idea here was simple: divide the total distance into an appropriate number of parts using the sum of the numerator and the denominator of the ratio, and then apportion the distances in inverse proportion to the speeds. Conceptually speaking, as argued earlier, the method is a reasonable one except that it did not work in the present case because the ratios of the walking and riding speeds were designed to be different.

**6. Trial and error A (brute force).** The use of trial and error was also fairly common, and the few groups that managed to solve the problem successfully all relied on trial and error. Two versions of trial and error emerged. The first one was what we refer to as the *brute force method* where groups would make an initial guess at the partition point, be it distance or time, typically the midpoint or the starting point, and then increment it systematically until they converged upon a solution.

**7. Trial and error B (connected to the ratios methods).** The second trial and error method (see Supplementary figure 2) was more sophisticated because it used information from the ratios method.

| **Ratios RSM** | **Multiple-variable algebraic RSM** |
| --- | --- |
| 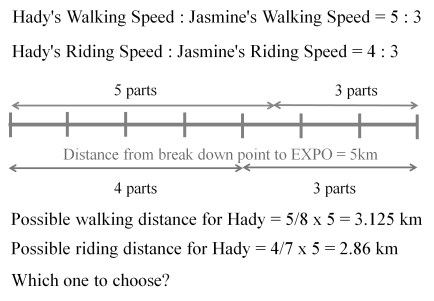 | 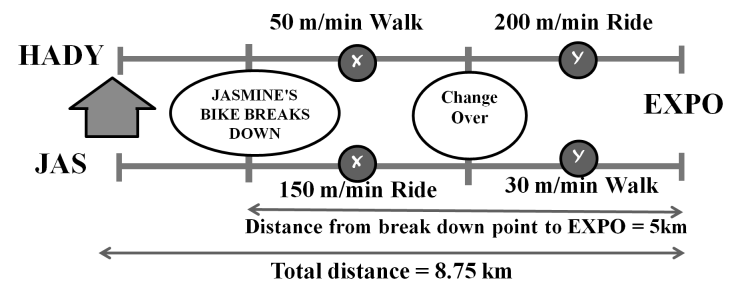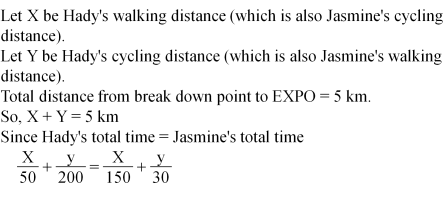 |
| **Trial & Error RSM**  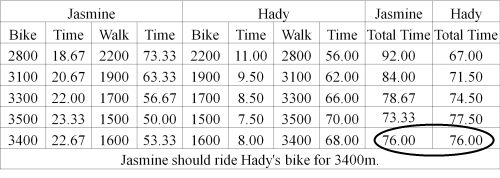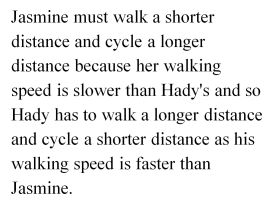 | |

*Supplementary figure 2*. Examples of the ratio, trial and error, and multiple-variable algebraic RSMs. RSM = representation and solution method. Actual RSMs produced by students were converted into stylized examples for easier reading.

Specifically, the choice of the starting guess for the partition point was informed by the partition distance derived from the ratio method. Therefore, instead of starting from 2.5 km as the initial guess as was the case in the brute force method, groups used the answer from the ratio method (either 2.8 km or 3.125 km) as the initial guess for the trial and error method. This reduced the computational and search load significantly and was consequently a faster method than the brute force method.

**8. Letter-symbolic algebra A (multiple variables)***.* A multiple-variable algebraic representation (as shown in Supplementary figure 2) was one of the two types of algebraic representations that the groups developed. However, there were more variables than equations, which made the system of equations unsolvable.

**9. Letter-symbolic algebra B (single variable)***.* A small proportion of groups was able to derive a single-variable algebraic representation of the problem. However, a lack of algebraic manipulation skills prevented them from being able to solve the equation successfully.

## 8. Student-generated RSMs to the Variance problem

A total of nine different RSMs emerged from this analysis (see Supplementary figures 3 and 4):

**1. Central tendencies.** This was the most common and usually the first strategy employed by the groups. Groups worked out the total, mean, median and/or mode for the three strikers. However, calculating total, mean and median did not yield an answer because those central tendencies were designed to be the same. Some groups used mode to say that because one striker has a bimodal distribution, he is the least consistent. Although this is not entirely incorrect, groups were unable to discern the more consistent striker between the remaining two strikers who had the same mode. Examples of the central tendencies methods can be found in Figure 3.

**2. Dot diagrams (aka histograms).** Groups also produced dot diagrams (see Supplementary figure 3) to get a sense of the data. They assert that since one striker has more goals bunched up around the mean than the other two, the striker is most consistent. Variants in this category include frequency counts, tables, polygons and stem-and-leaf diagrams.

**3. Trend lines (aka line graphs).** As shown in Supplementary figure 3, some groups tried drawing trend lines for the three strikers, maintaining that the one which has fewer rises and drops is the most consistent.

| **Central tendencies and Range RSMs**  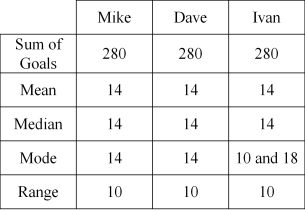 | **Dot diagram RSM**  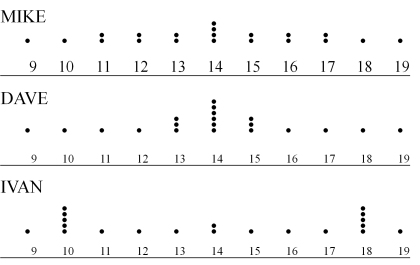 |
| --- | --- |
| **Trendlines RSM**  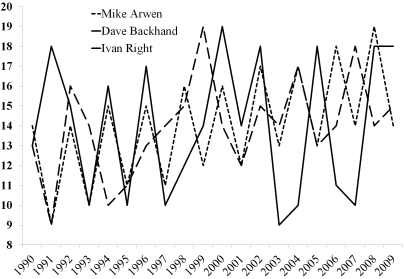 | **Counting and Ranking RSMs**  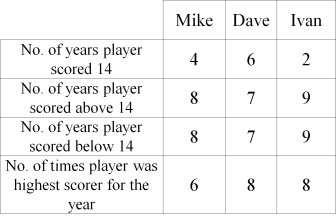 |

*Supplementary figure 3*. Examples of the central tendencies, range, dot diagram, trend lines, counting and ranking RSMs. RSM = representation and solution method. Actual RSMs produced by students were converted into stylized examples for easier reading.

**4. Counting.** The use of counting was relatively common. Groups counted the number of years the striker scored at the mean, concluding that the more goals at the mean, the more consistent the striker. A variation of this method is *counting around the mean*. Here, groups also counted the number of years the strikers scored around the mean (for example, 13–15, or 12–16) and, by applying the same reasoning, conclude that the more goals around the mean, the more consistent the striker. Another variation of this method is *counting away from the mean relative to the mean*. Groups counted the number of years the strikers scored at the mean relative to the number of years the strikers scored away from the mean, reasoning that the higher the ratio, the more consistent the striker. Examples and variants of the counting method are shown in Supplementary figure 3.

**5. Ranking.** Still others ranked the strikers based on how many years the strikers scored the highest number of goals with the reasoning that the higher this number, the more consistent the striker (see Supplementary figure 3). In this case, students might have confused the concept of “good” with “consistent”, thinking that if someone is good, he is also consistent and vice versa. During the consolidation phase of PF, the teachers make it a point to address this misconception and distinguish the concepts from each other.

**6. Range.** Range was the first of the two more commonly produced deviations methods. Here, the groups took the maximum number of goals and subtracted it from the minimum number of goals. However, this strategy did not work because we have designed the problem such that the ranges were all the same.

**7. Year-on-year deviations, sum, with signs.** The year-on-year deviations method was the other common deviation method that groups used. Groups took deviations from one year to the next and summed the differences with signs (see Supplementary figure 4). Their argument was that the striker with the least deviations was the more consistent one. However, this method is problematic as the signs cancel out, giving the impression that the deviations are smaller than they really are. There were two variations of this method. First, some groups were aware of the issue of different sample sizes affecting their measure. So, they went on to average the deviations. Second, there were groups that simply identified the year-on-year deviations without summing or averaging. Such groups eyeball the differences and argue that the smaller the differences between years, the more consistent.

| **Year-on-year deviations RSM** |
| --- |
| 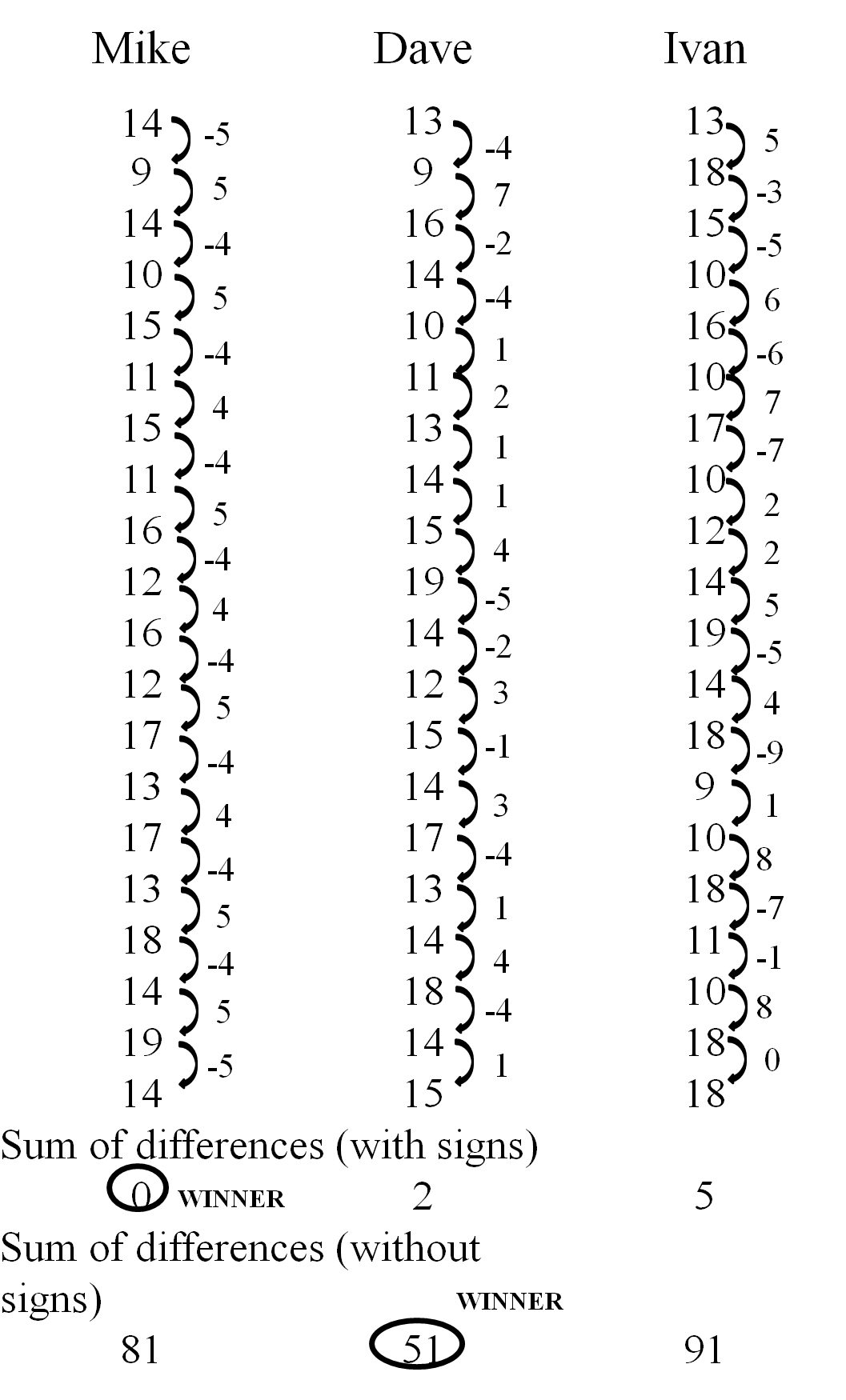 |

*Supplementary figure 4*. RSMs of the year-on-year deviations, summed with signs and without signs. RSM = representation and solution method. Actual RSMs produced by students were converted into stylized examples for easier reading.

**8. Absolute year-on-year deviations, summed.** To counter the cancelling out problem in the previous method, some groups take the sum of absolute year-on-year deviations instead of leaving the signs intact (again, see Supplementary figure 4). Conceptually speaking, this method is reasonable if the data were temporally dependent. However, since the data in the problem were not temporally dependent, this measure of deviation runs into the problem of being order-sensitive. This means that if the data were to be rearranged, taking year-on-year deviations would yield a different number the second time around. Once again, there were two variants of this method: the average and the eyeballing method.

**9. Deviations from the mean.** A small proportion of groups took the deviations from a fixed point, usually the mean and sometimes the median, although they were unable to express clearly why they chose that particular point of reference. Deviations from the mean add up to zero, which was sometimes noticed by the students.
